# Supplementary material for: A modular fluorescent camera unit for wound imaging
Source: Commun Biol. 2025 Jul 5;8:1010. doi: 10.1038/s42003-025-08423-y (PMC12228714; doi:10.1038/s42003-025-08423-y)
Supplement: Supplementary file 2 — Supplementary Information [file 42003_2025_8423_MOESM2_ESM.pdf]

Supplementary Information
A modular fluorescent camera unit for wound imaging

Maryam Tebyani[1,2,*], Gordon Keller[1], Wan Shen Hee[1], Prabhat Baniya[1], Alex Spaeth[1,2], Tiffany Nguyen[1], Harika Dechiraju[1], Anthony Gallegos[3], Héctor Carrión[4], Derek Hamersly[5], Cristian Hernandez[1], Alexie Barbee[1], Hao-Chieh Hsieh[1], Elham Aslankoohi[1], Hsin-ya Yang[3], Narges Norouzi[6], Min Zhao[3,7], Alexander Sher[5], Rivkah R. Isseroff[3], Marco Rolandi[1,2], Mircea Teodorescu[1,2]
[1]Department of Electrical and Computer Engineering, University of California Santa Cruz, Santa Cruz, California, 95064, USA
[2]Genomics Institute, University of California Santa Cruz, Santa Cruz, California, 95060, USA
[3]Department of Dermatology, School of Medicine, University of California Davis, Sacramento, California, 95816, USA
[4]Department of Computer Science and Engineering, University of California Santa Cruz, Santa Cruz, California, 95064, USA
[5]Santa Cruz Institute for Particle Physics, University of California Santa Cruz, Santa Cruz, California, 95060, USA
[6]Department of Electrical Engineering and Computer Science, University of California Berkeley, California, 94720, USA
[7]Department of Ophthalmology & Vision Science, University of California Davis, Sacramento, California, 95817, USA
[*]Corresponding authors: mtebyani@ucsc.edu, mrolandi@ucsc.edu, mteodore@ucsc.edu

Supplementary figures, tables, and text

Imaging Unit Calibration and Components

We use the USAF 1951 Resolution Target from Edmund Optics to evaluate the resolution of the Imaging Unit. Figure S1 shows the calibration slide imaged with the configuration shown in Figure S2b. The image is a raw Bayer mask and appears green due to the higher proportion of green pixels in Bayer filters. The resolution slide is based on line pairs per millimeter (lp/mm). The line pairs in Group 4, Element 3 are still discernible, after which the lines begin to blur together. This element has 20.16 lp/mm, giving a line pair width of 49.60 µm and a single line width of 24.80 µm. Therefore, this configuration (shown in Figure S2a, can resolve features down to 24.80 µm.

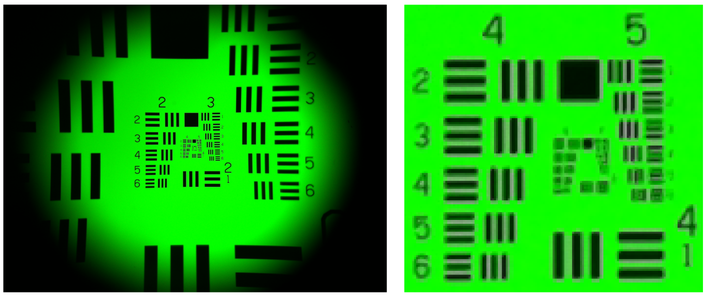

Fig. S1: Raw Bayer mask image (green due to the higher proportion of green pixels in Bayer filters) of a calibration slide to showcase the resolution of the imaging unit. The full image includes the 6mm diameter field-of-view, and the zoomed-in image shows the smallest resolvable lines on the slide.

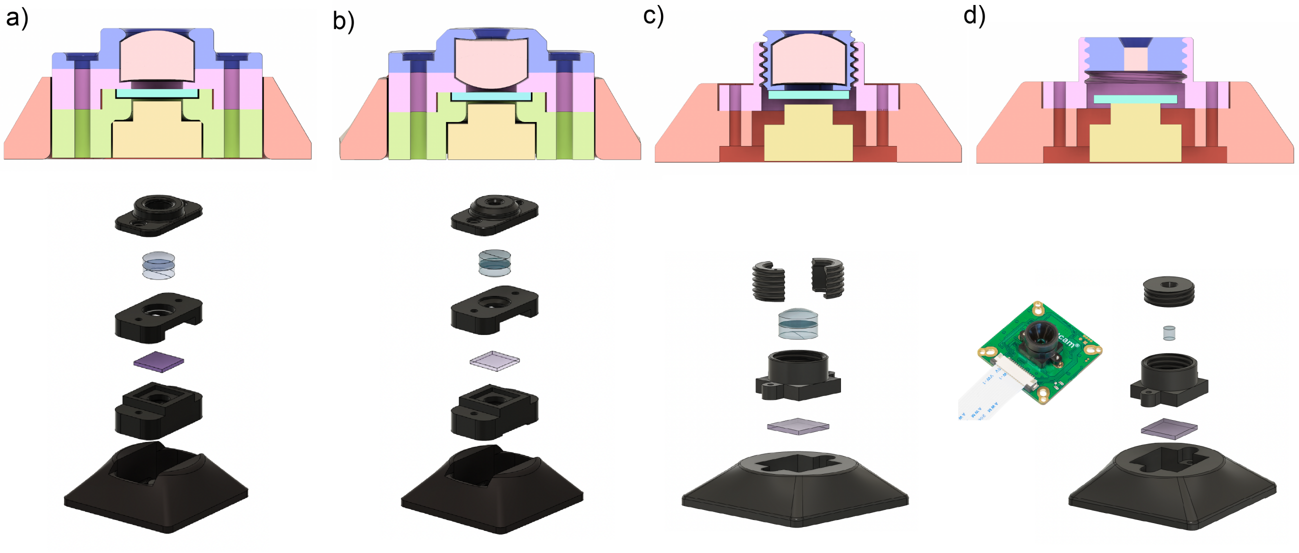

Fig. S2: CAD models of the various configurations of the imaging unit used in the paper. The cross-sectional view shows the optical configuration, and the expanded view shows the individual components. a) Configuration used for in vivo NO detection experiment. b) Configuration used for all pH detection experiments. Uses the same hardware as the configuration in (a) with the lens inverted. c) Configuration used for THP-1 cell imaging. This alternative mount design features a threaded lens insert that can be inverted to switch between configurations (a) and (b). d) Configuration used for macrophage (M1 Type) imaging. Uses a commercial mount and stock lens. The CAD model of the lens and threaded features on the lens mount are approximations, as there are no available CAD models for these components.

The Imaging Unit is comprised of 3D printed and commercial parts. Figure S2 shows the various camera configurations used in the presented experiments. All configurations use the same camera and basic structure, including a filter, lens, along with a 3D printed shroud and holders. The top row shows a cross-section of the configuration, and below is an exploded view of the components. Figure S2a was used for the in vivo NO detection, Figure S2b was used for the pH experiments, Figure S2c was used for THP-1 cell imaging, and Figure S2d was used for macrophage (M1) imaging. Figure S2c features a threaded lens insert to allow for changing between configurations a and b, but this design feature was not thoroughly explored in this work.

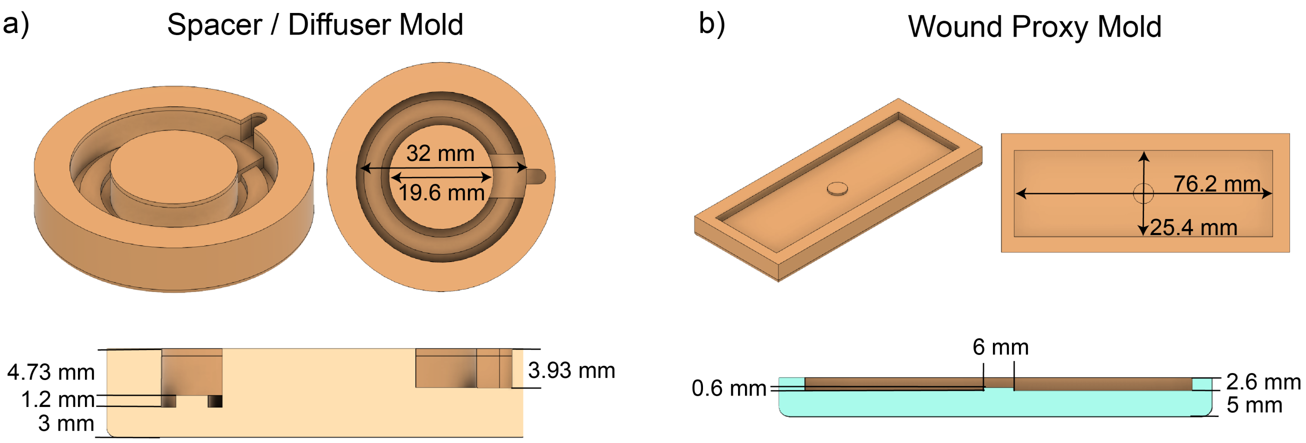

Fig. S3: CAD models of 3D printed molds to fabricate PDMS parts. a) The Spacer/Diffuser mold for the imaging unit. b) The wound proxy mold for pH calibration.

Figure S3 shows the two 3D printed molds used for the PDMS casting process in this work. Figure S3a shows the mold for the spacer/diffuser component of the imaging unit. The depth shown was used for the pH experiments and can be altered to meet various imaging requirements. Figure S3b shows the 3D printed mold for the wound proxy used in the pH experiment to calibrate the Snarf dye response.

Table S1 shows the BoM for the imaging unit, Table S2 shows the BoM for the peripherals and the computing unit, and Table S3 shows the BoM for the LED PCB.

**Component**	**Part Name**	**Manufacturer**	**Price (USD)**
Lens	9mm Diameter x 12mm EFL Aspherized Achromatic	Edmund Optics	\$118
Camera	21MP IMX230	Arducam	\$37
Bandpass Filter (Snarf)	645nm FWHM 17nm Bandpass Filter	PIXELTEQ (10x10mm)	\$86
Bandpass Filter (DAF-FM, Calcein AM)	515nm FWHM 10nm Bandpass Filter	PIXELTEQ (10x10mm)	\$86
Microcontroller	Raspberry Pi 3 Model B	Raspberry Pi	\$35
M3 x 5mm Screw	Stainless Steel Pan Head Phillips Screw (90116A150)	-	-
M3 Threaded Inserts	Brass Heat-Set Inserts for Plastic (94459A130)	-	-
M2 x 16mm Screw	Stainless Steel Pan Head Phillips Screws (95836A281)	-	-
M2 Nut	Steel Hex Nut (90592A004)	-	-
Lens & Mount (Macrophage Imaging)	B0277 M12 Low distortion lens EFL: 4.73mm, FoV(H): 50°, F/NO:2.2	Arducam	-
Flexible Ribbon Cable	30cm Ribbon Flex Extension Cable	Arducam	\$5.00

Table S1: Bill of Materials for the Imaging Unit, not including the LED PCB components.

Component	Part Name	Manufacturer	Price (USD)
Raspberry Pi	Raspberry Pi 3 Model B	Raspberry Pi	$35.00
Monitor	-	-	-
Keyboard	-	-	-
Mouse	-	-	-
Monitor Cable	3ft Mini HDMI to HDMI Adapter Cable	-	-

Table S2: Bill of Materials for the peripherals and the computing unit.

Component	Part Name	Manufacturer	Price (USD)
LED PCB	3-bank variable brightness LED PCB ring	PCBWay	-
520nm LED (Snarf)	QBLP595-IG	QT Brightek	$0.38
496nm LED (DAF-FM, Calcein)	SMLD12E3N1WT86	Rohm Semiconductor	$0.74
White LED (Brightfield)	QBLP595-IW-2897	QT Brightek	$0.39
NPN Transistor	MMBT2222A-TP	Micro Commercial Co	$0.10
200Ω Resistor (LED)	ERJ-2GEJ201X	Panasonic Electronic Components	$0.10
200Ω Resistor (Transistor)	RK73B1JTTD201J	KOA Speer Electronics, Inc.	$0.10
FPC Connector	FH33-6S-0.5SH(10)	Hirose Electric Co Ltd	$1.44
Flexible Ribbon Cable	6 Pins 0.5mm Pitch 150mm FPC FFC	uxcell	$5.49
FPC Adapter Board	FFC FPC 6 Pin 0.5mm 1mm Pitch to DIP 2.0mm	uxcell	$5.49
Jumper Wires	-	-	-

Table S3: Bill of Materials for LED PCB components.

pH Imaging

Figure S4 shows the calibration results for characterizing the fluorescent response to Snarf. A commercial microscope (Keyence) was used with 100% excitation and 1/2 sec exposure. The Imaging Unit is used with green LEDs installed on the LED PCB and 1/2 sec exposure. The calibration results for the commercial microscope are used in accordance with the technical specifications for Snarf. The lower wavelength band (590nm) shows a negative linear response between pH 6 to pH 9, and the higher wavelength (640nm) shows a positive linear response between pH 5 and pH 9. Similarly, the imaging unit shows a positive linear response between pH 5 and pH 9 at the higher wavelength. We use the ratiometric calibration to determine the pH model for the commercial microscope, which is given by

$$\begin{aligned}
\mathrm{pH} &= \mathrm{pK_A} + \log\left(\frac{R - R_a}{R_b - R}\right) \\
R &= \mathrm{FI}_{\lambda=640}/\mathrm{FI}_{\lambda=590} \\
R_a &= \min(R) \\
R_b &= \max(R)
\end{aligned}$$

where $\mathrm{pK_A}$ is 7.5, and FI is the fluorescent intensity of pH 5 through pH 9, as excited at wavelength λ equal to 640 nm or 590 nm.

Figure S4 shows the calibration for the commercial microscope and the imaging unit using Snarf dye diluted in pH buffer, pipette in the well of the PDMS wound proxy. Figure S4a shows the fluorescence response of the two imaging systems. We observe that at pH 4, both the commercial microscope and our imaging unit show higher fluorescent intensity than at pH

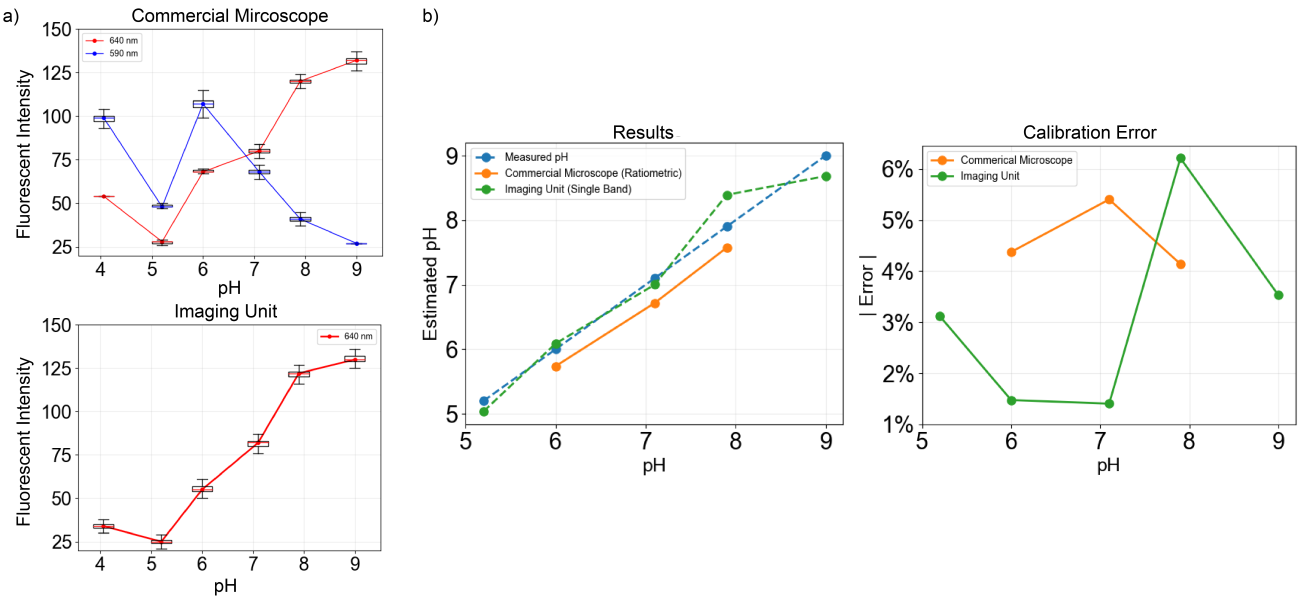

Fig. S4: Snarf calibration for pH detection. a) The fluorescent intensity of samples ranging from pH 4 to pH 9 using a commercial fluorescent microscope and the imaging unit. The commercial microscope images use both wavelengths (590nm and 640nm), while the imaging unit uses a single band at 645nm. The distribution of the fluorescent intensity (Commercial Microscope n=27,648, Imaging Unit n=858,407) is shown as box plots, where the center line denotes the median, the box spans the interquartile range (IQR), and the whiskers extend to the most extreme data points within 1.5xIQR. b) Results: The measured pH of the calibration sample in the valid range (pH 5-9) is plotted against the estimated pH using the standard calibration method with the commercial microscope, and a linear model using the single-band imaging unit. Calibration Error: The magnitude of the error between the calibrated and measured pH values using the commercial microscope and our imaging unit is plotted for each pH in the valid range.

5 when measured at the 640 nm wavelength. Based on this non-linear response at pH 4, we established pH 5-9 as our valid calibration range. The Results plot in Figure S4b shows the pH of the buffer as measured by a benchtop meter on the x-axis and the estimated pH using the corresponding calibration on the y-axis. The mean fluorescent intensity from the Imaging Unit calibration is used for the pH mapping of the in vivo wounds in the main text. The calibration error plot shows the percentage error magnitude between the estimated and the measured pH. The calibration error is also reported in the main text in Table 1.

Using the calibration, we performed an experiment to estimate the pH of in vivo wound beds. We used k-means clustering to visualize various regions of pH within the wound bed. To determine the number of clusters to use, we made an elbow plot that visualizes the sum of squared distances (SSD) of each pixel from cluster means as a function of the number of clusters, k. This elbow plot, shown in Figure S5 is a popular method of determining the optimal number of clusters to use in a k-means analysis. We find that there is a significant drop at k=2 and another drop at k=5. The analysis was limited to k=8 since the SSD values level off, indicating that additional clusters do not significantly enhance the model. This trend remains stable across the 11 Z-stack images. We use k=5 in the presented analysis.

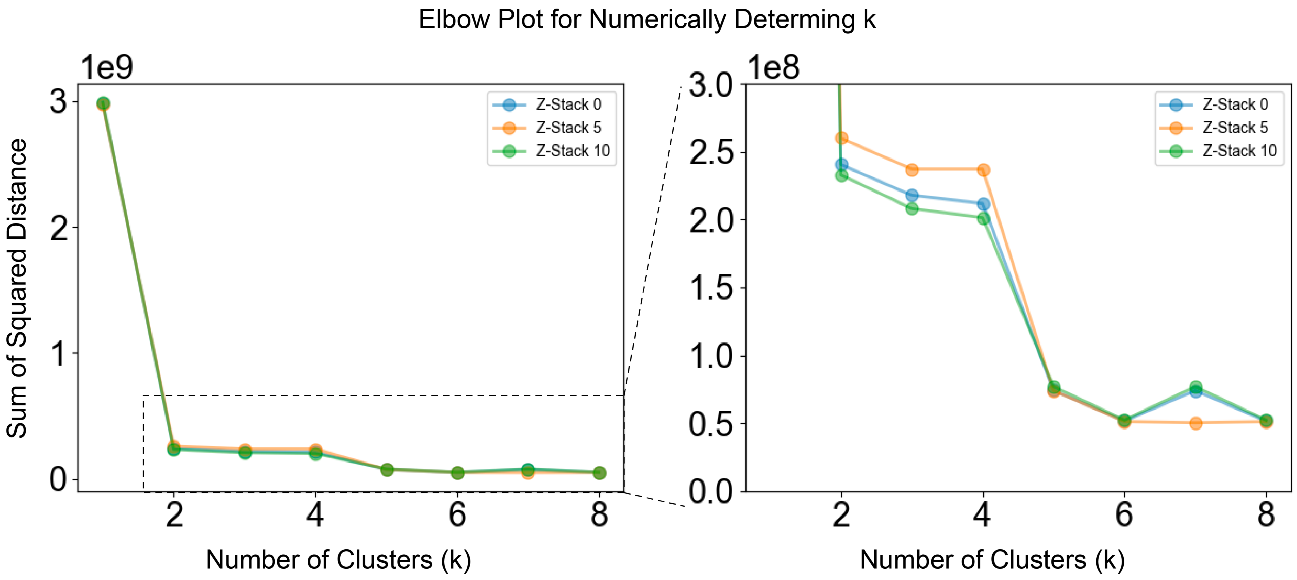

Fig. S5: K-means analysis for pH detection in vivo. This figure shows the elbow plot and zoomed-in elbow plot used to determine the optimal number of clusters (k) for the k-means algorithm. The plot shows a sharp decrease in the Sum of Squared Distances (SSD) at k=2, with another significant drop at k=5. Of 11 available Z-Stack images, the plot shows the results for Z-Stack Images 0, 5, and 10.

NO and Brightfield Imaging

To demonstrate the use of the imaging unit to investigate the wound healing process, we captured both brightfield and fluorescent NO wound images, as reported in the main text. These images are shown in Figure S6. The fluorescent images were dyed with DAF-FM to highlight NO presence. The brightfield images were processed using the HealNet pre-trained wound stage prediction model, while fluorescent images were used to correlate NO levels with the healing day and percent re-epithelialization (% re-epi.) using a Ridge regression model.

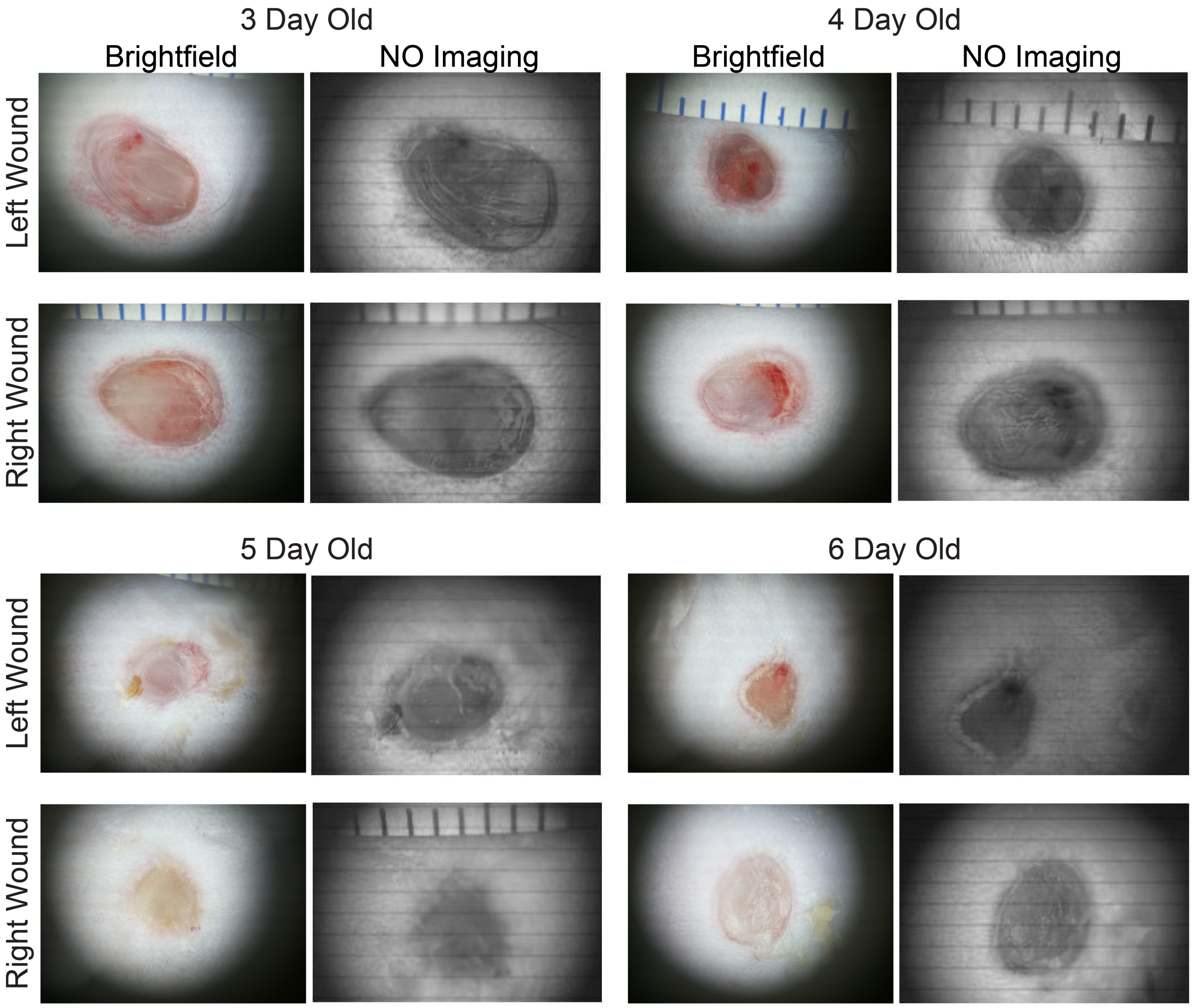

Fig. S6: Brightfield and fluorescent NO pictures captured with our imaging unit. Fluorescent images are dyed with DAF-FM for nitric oxide (NO) imaging.

Healnet Model Usage

To enable Healnet to predict wound stages using images captured by the imaging unit, we perform a color-matching procedure to align the color balance of the imaging unit with those used for Healnet training images. Healnet is trained with wound images captured by an iPhone. Using the color histogram of a Healnet iPhone reference image, we observe that the reference images appear warmer, with skin tones more red than pale white, compared to the imaging unit. The Healnet reference image and a sample imaging unit image are shown in Figure S7a and b, along with the corresponding color histograms.

To match the color balance of the reference images with the new images, the overall average color values of the reference images are divided by the average color values of the new images to come up with RGB correction factors: [1.09, 0.80, 0.72]. After applying this transformation to each respective color channel, the new imaging unit images appear much warmer and more closely resemble the Healnet reference images, as shown in Figure S7c. Next, circle crops that HealNet expects were applied to focus on the wound bed, which removes the spotlight effect present in the original images, shown in Figure S7c. Finally, the wound stage probability was predicted using HealNet. The results, reported in the main text, seem to follow visual wound features. For example, the 3-day-old large red Right Wound in Figure S6 is detected as hemostasis. Meanwhile, the smaller dry wound on the 6-day-old Left Wound is detected as proliferation.

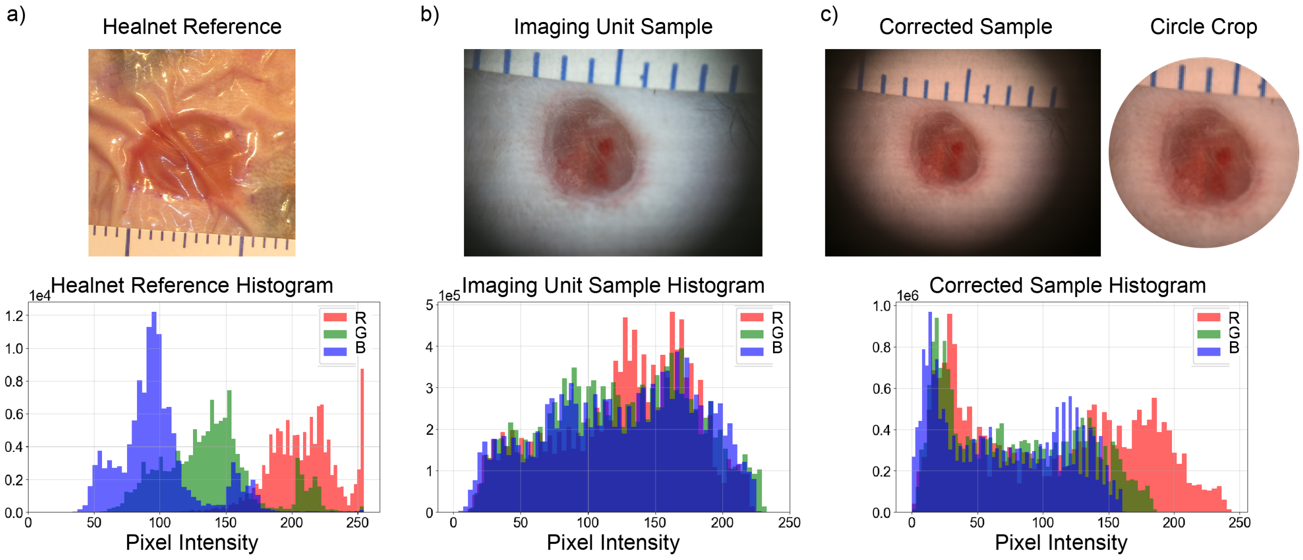

Fig. S7: Healnet image pre-processing. This figure shows the color balance matching and cropping for Healnet model usage with the imaging unit. a) A Healnet reference image and a frequency histogram for the RGB color values of the Healnet reference. b) A sample brightfield wound image from the imaging unit and the frequency histogram for the RGB color values. c) The Corrected Sample image is the color-corrected imaging unit sample, and the Circle Crop image is the final cropped input to the Healnet model. The histogram is shown for the uncropped corrected sample.

NO Model Cross Validation

To determine the stability and validity of the NO Ridge regression, we performed cross-validation with the 8 fluorescent NO images. The model is trained using normalized values for the Day and % re-epi. data. Figure S8 shows the resulting models of each fold for the full wound model. We see that while there is variation in the coefficients, the general trends of the coefficients are stable, indicating the robustness of the model.

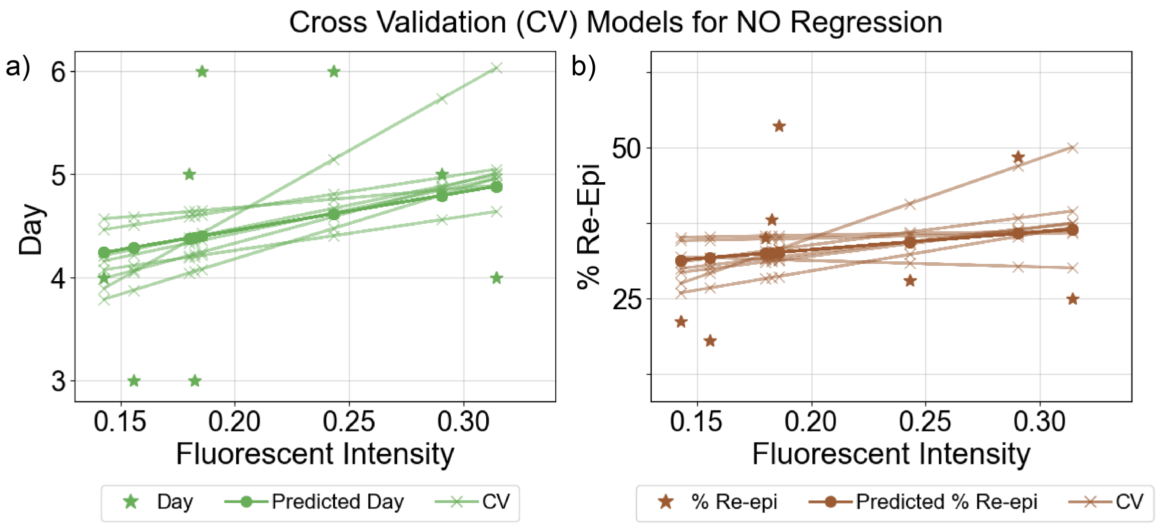

Fig. S8: Model stability in cross-validation (CV) analysis of NO linear model (Full Wound - Fluorescent Intensity) predicting day and percent re-epithelialization (% re-epi). The plots show the measured data, the predictions from training on all measured data points, and the model outputs from the 8-fold CV, where each fold corresponds to leaving out sample k, and k is 1 of 8 samples. a) The Day, the full model Predicted Day output, and the CV model outputs, as a function of the fluorescent intensity. b) The measured % re-epi, the full model Predicted % re-epi output, and the CV model outputs, as a function of the fluorescent intensity.

Imaging Unit Diagrams & Assembly

Figure S9a shows the connection between our system's peripherals, computing unit or microcontroller, and the Imaging Unit. Figure S9b shows the circuit diagram for the LED PCB. The LED PCB hosts 3 LED banks which can be independently addressed through the microcontroller's GPIO:DAC pins through Driver FETs.

Figure S9c shows the connections between the peripherals, computing unit, and imaging unit. The peripherals provide user input to the microcontroller and real-time image viewing from the Imaging Unit. The GPIO pins control the LED banks through a PWM signal. Finally, the microcontroller sends image capture protocols to the Imaging Unit and receives image files in data formats .jpg and .h264. We use the libcamera library to capture images. In particular, we use libcamera-still to capture images at various focal lengths and libcamera-vid for real-time viewing as needed to ensure the sample is correctly positioned in the frame.

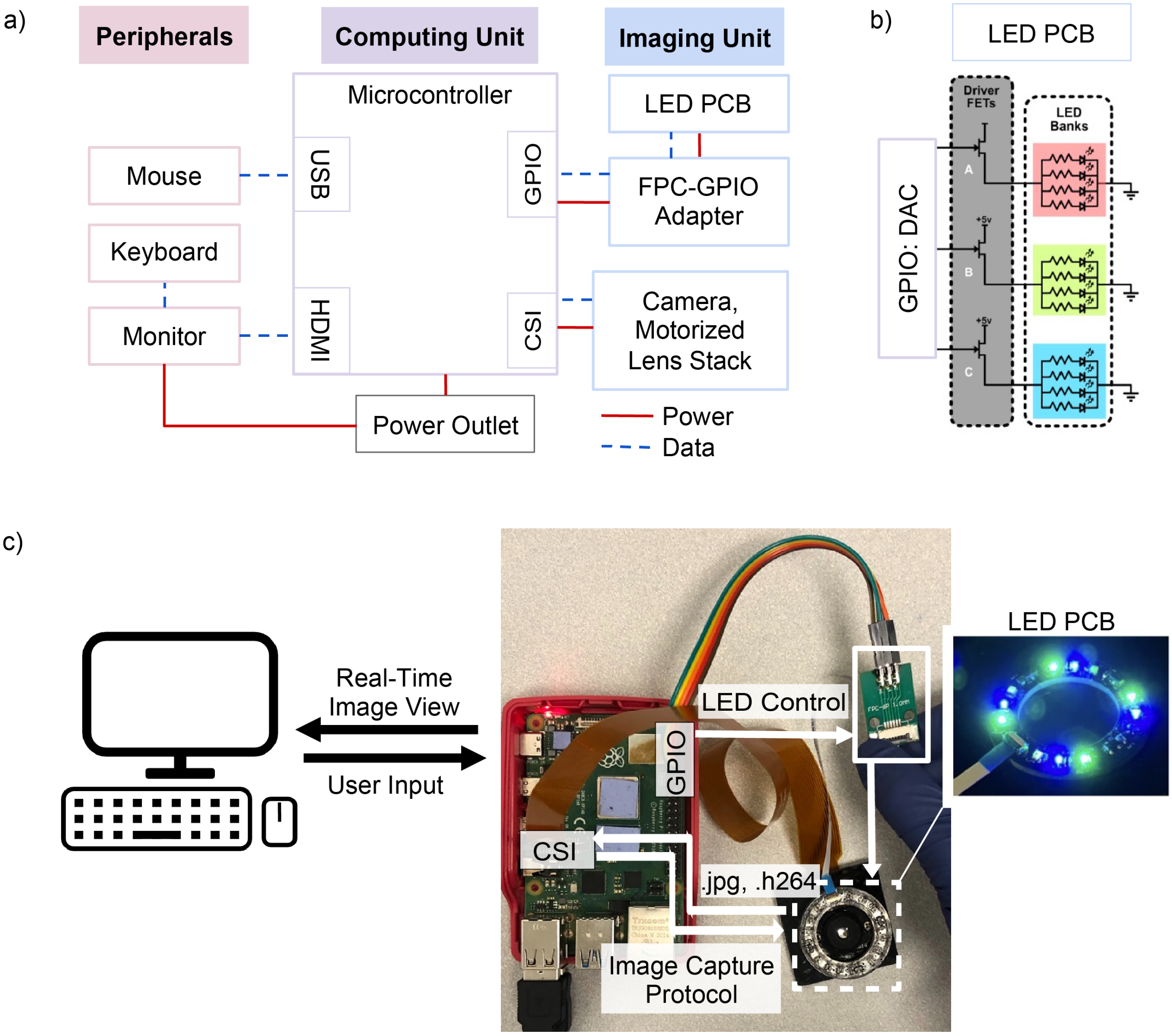

Fig. S9: a) This diagram describes the electrical connections for operating the Imaging Unit. The Imaging Unit camera uses a ribbon cable for power and data communication with the microcontroller via the Camera Serial Interface (CSI). The LED PCB uses a flexible printed circuit (FPC) ribbon cable which connects to an FPC-GPIO Adapter board with socket headers that connect to the microcontroller GPIO pinouts. Peripherals include a monitor, keyboard and mouse. b) This circuit diagram describes the LED PCB. The LED PCB provides the option for up to 3 LED colors (LED banks), with 4 LEDs per color. c) This diagram describes the data sent between the peripherals, computing unit, and imaging unit. The image of the LED PCB demonstrates the use of 2 LED colors.

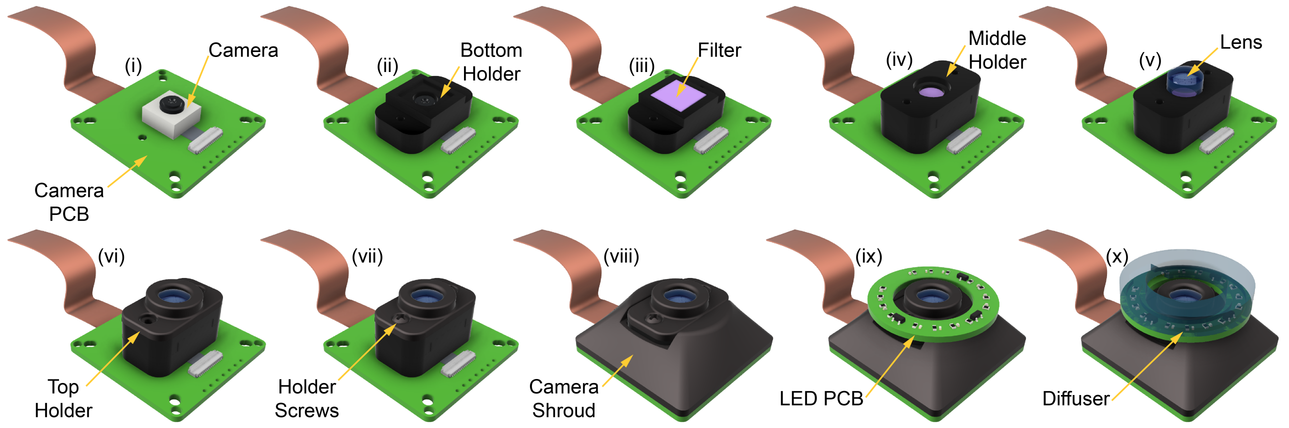

Fig. S10: Imaging Unit assembly steps. An Arducam 21 MP camera (CMOS Sensor and CMOS PCB) is used as the base of the camera, and an optional fluorescent filter, additional optical lens, LED PCB, and diffuser are held in place with 3D-printed mounts.

Figure S10 shows the steps to assemble the Imaging Unit. The Arducam camera comes mounted to a Camera breakout board. On top of this board, we place the bottom holder, which has a cut-out to hold a 10x10mm filter. The middle holder has an indentation for the lens, and finally, the top holder sits on top of the lens. The mount is secured with 2 holder screws. Next, a camera shroud slides through the mount to sit on top of the camera breakout board and is also held by screws. The LED PCB is glued onto the camera shroud. During use, the diffuser is placed on top of the LED PCB.

Figure S11 shows the LED PCB layout diagram, including the LEDs, driver FETs, resistors, and flexible ribbon cable connector. These components are listed in the LED PCB Bill of Materials (BoM), Table S3. The Top surface of the LED PCB interfaces with a matching groove in the spacer/diffuser, while the bottom surface sits on top of the 3D-printed shroud.

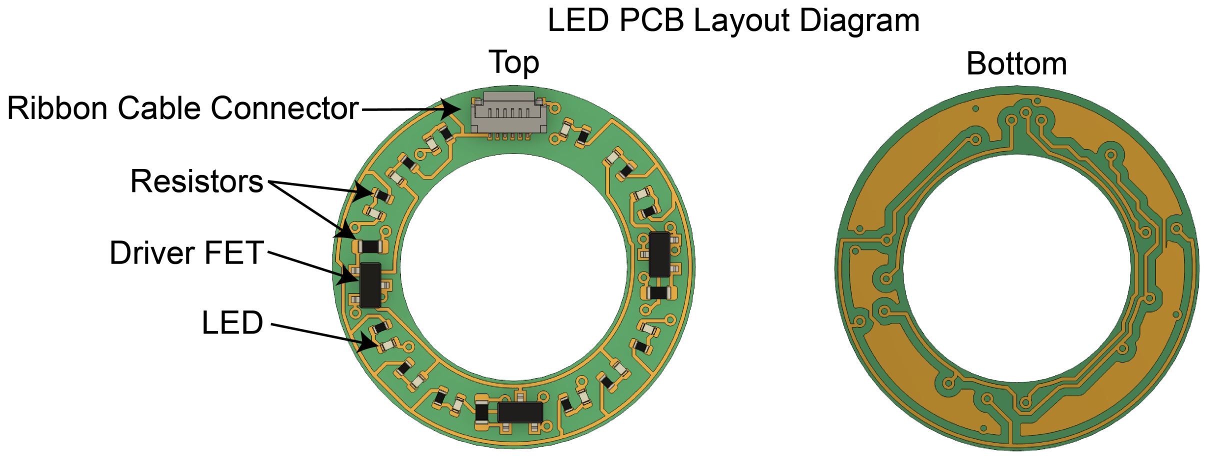

Fig. S11: Top and bottom of the LED PCB, showing traces for the Flexible Printed Circuit (FPC) ribbon cable connector, resistors, driver FETs, and LEDs.

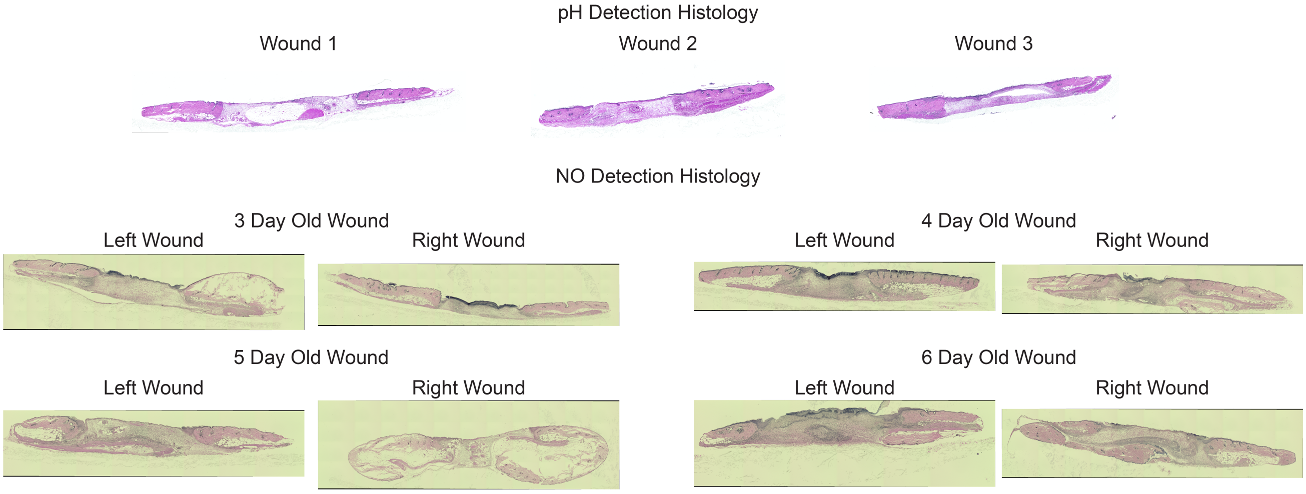

Fig. S12: Histological samples for in vivo pH and NO detection experiments.

Histological Analysis

Figure S12 shows the histological samples for the pH and NO experimental wounds used to quantify the % re-epi. This data is reported for the pH experiment and used to train the regression model for the prediction of re-epithelialization in the NO experiment. First, the left and right lengths of the epithelial tongues on each side of the wound are measured in micrometers. The center distance between the innermost tips of the tongues, the part of the wound bed that is not yet epithelialized, is also measured. The total width of the wound is defined as a sum of the left, right, and center distances. The % re-epi. is calculated as

$$\%\ \text{re-epi.} = \frac{\text{left} + \text{right}}{\text{total width}} * 100$$
